# Supplementary material for: Causal interplay between lactose intolerance and gut microbiota: a combined bidirectional Mendelian randomization and in vivo validation study
Source: Front Nutr. 2026 Jun 1;13:1803337. doi: 10.3389/fnut.2026.1803337 (PMC13265576; doi:10.3389/fnut.2026.1803337)
Supplement: Supplementary file 1 [file Data_Sheet_1.zip › supplementary materials/Forward/forest plot/ebi-a-GCST90027656.finngen_R12_E4_LACTONAS.pdf]

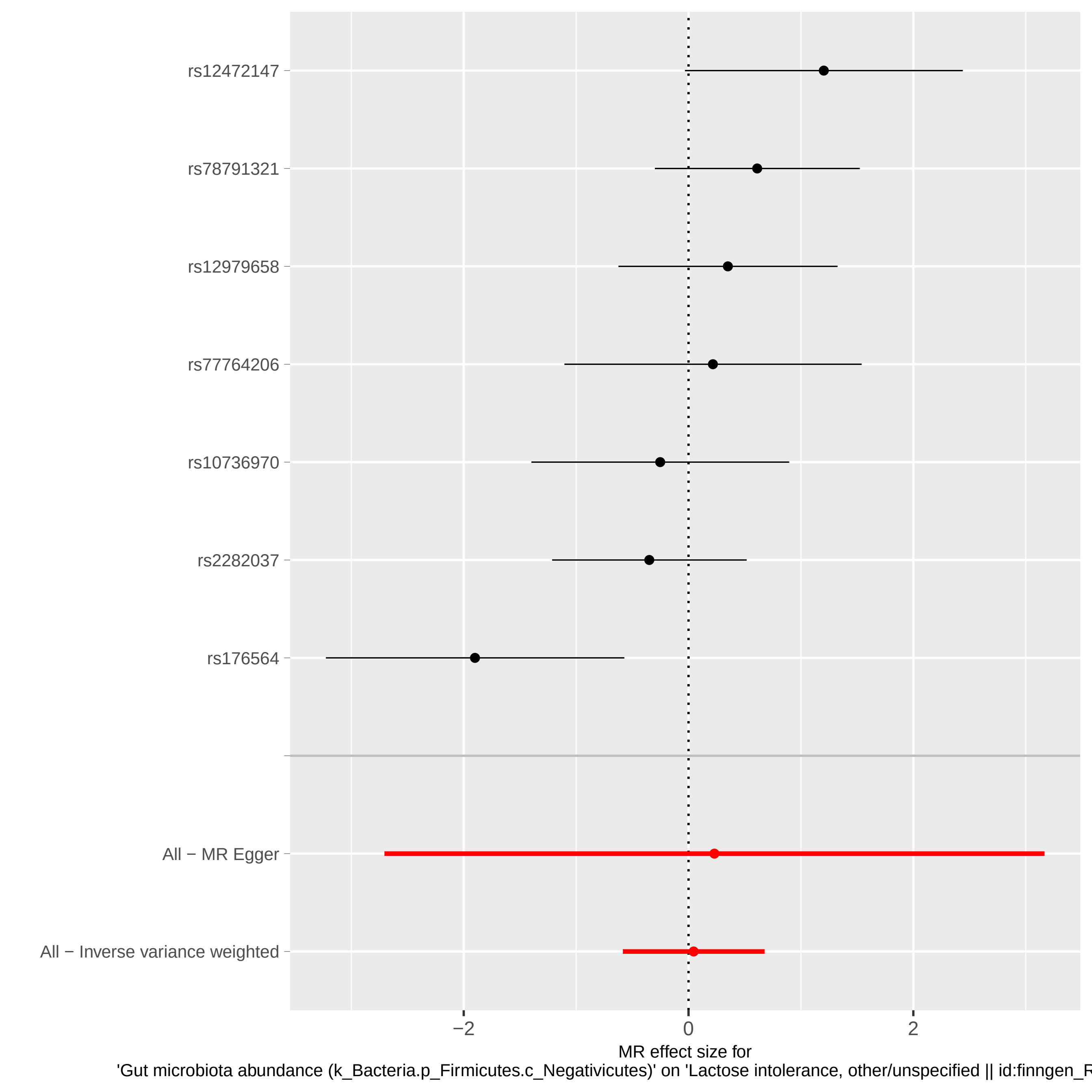
rs12472147
rs78791321
rs12979658
rs77764206
rs10736970
rs2282037
rs176564
All − MR Egger
All − Inverse variance weighted
−2
0
2
MR effect size for
'Gut microbiota abundance (k_Bacteria.p_Firmicutes.c_Negativicutes)' on 'Lactose intolerance, other/unspecified || id:finngen_R
